# The Rapid Assessment of Avoidable Blindness survey: Review of the methodology and protocol for the seventh version (RAAB7)

**DOI:** 10.12688/wellcomeopenres.20907.1

**Published:** 2024-03-08

**Authors:** Ian McCormick, Robert Butcher, Jacqueline Ramke, Nigel M Bolster, Hans Limburg, Hannah Chroston, Andrew Bastawrous, Matthew J Burton, Islay Mactaggart

**Affiliations:** 1 International Centre for Eye Health, London School of Hygiene & Tropical Medicine, London, UK; 2 Clinical Research Department, London School of Hygiene & Tropical Medicine, London, UK; 3 School of Optometry and Vision Science, The University of Auckland, Auckland, New Zealand; 4 Peek Vision, London, UK; 5 Independent consultant, Grootebroek, The Netherlands; 6 National Institute for Health Research Biomedical Research Centre for Ophthalmology at Moorfields Eye Hospital NHS Foundation Trust and UCL Institute of Ophthalmology, London, UK

**Keywords:** Vision impairment, blindness, cataract, effective coverage, population survey, mobile data collection, open data, data repository

## Abstract

The Rapid Assessment of Avoidable Blindness (RAAB) is a population-based cross-sectional survey methodology used to collect data on the prevalence of vision impairment and its causes and eye care service indicators among the population 50 years and older. RAAB has been used for over 20 years with modifications to the protocol over time reflected in changing version numbers; this paper describes the latest version of the methodology–RAAB7. RAAB7 is a collaborative project between the International Centre for Eye Health and Peek Vision with guidance from a steering group of global eye health stakeholders. We have fully digitised RAAB, allowing for fast, accurate and secure data collection. A bespoke Android mobile application automatically synchronises data to a secure Amazon Web Services virtual private cloud when devices are online so users can monitor data collection in real-time. Vision is screened using Peek Vision’s digital visual acuity test for mobile devices and uncorrected, corrected and pinhole visual acuity are collected. An optional module on Disability is available. We have rebuilt the RAAB data repository as the end point of RAAB7’s digital data workflow, including a front-end website to access the past 20 years of RAAB surveys worldwide. This website ( https://www.raab.world) hosts open access RAAB data to support the advocacy and research efforts of the global eye health community. Active research sub-projects are finalising three new components in 2024-2025: 1) Near vision screening to address data gaps on near vision impairment and effective refractive error coverage; 2) an optional Health Economics module to assess the affordability of eye care services and productivity losses associated with vision impairment; 3) an optional Health Systems data collection module to support RAAB’s primary aim to inform eye health service planning by supporting users to integrate eye care facility data with population data.

## Plain language summary

In 2020 there were an estimated 1.1 billion people with vision impairment globally. Vision impairment negatively affects people’s quality of life, social inclusion and productivity. The Rapid Assessment of Avoidable Blindness (RAAB) survey tool collects information about the vision and eye health of people aged 50 years and older in a defined population. It has been used worldwide for over 20 years to inform eye health service planning. This paper outlines the current survey methodology and summarises recent and upcoming developments. The RAAB project team has updated the survey to allow users to measure vision and collect other information on mobile devices (telephones or tablets) and send the findings directly to a central computer for automated analysis. The project team has built a new website to store this information and to allow anyone interested to find out more about the surveys done to date. The RAAB project continues to develop new features to make the information collected in surveys more useful for eye health service planning and eye health advocacy.

## Introduction

The Rapid Assessment of Avoidable Blindness (RAAB) is a population-based cross-sectional survey methodology used to collect data on the prevalence of vision impairment and its causes and eye care service indicators among the population 50 years and older in a defined geographic area.

In 1995, Dr Hans Limburg and colleagues in India developed a cluster random sampling survey protocol to estimate the prevalence of cataract blindness at the district level, where blindness prevention strategies were planned and carried out. The methodology was designed to provide cataract blindness prevalence estimates with reasonable precision and to be simple and affordable enough to be repeated at 5-year intervals to monitor the effect of eye care interventions ^1^. In 2001, a manual based on this survey protocol–and an accompanying software package for data entry and analysis–was published as the Rapid Assessment of Cataract Surgical Services (RACSS) ^2^.

The International Centre for Eye Health (ICEH) is a global eye health research and education group based at the London School of Hygiene & Tropical Medicine (LSHTM). In 2006, a modified version of RACSS was described as the Rapid Assessment of Avoidable Blindness, or RAAB, survey methodology ^3^. Minor changes in RAAB protocol over time have been reflected in changes to the version number, from RAAB4.02 (2005), RAAB4.03 (2008), RAAB5 (2013) through to RAAB6 (2013). In 2015, a pilot mobile application (mRAAB6) was introduced to improve the speed and accuracy of data collection compared to paper questionnaires. Development of RAAB7 started in 2018 as a collaboration between ICEH and Peek Vision, a not-for-profit social enterprise that develops software and a data intelligence platform for eye care providers to deliver more effective and equitable screening programmes and rapid surveys, including RAAB7 and the School Eye Health Rapid Assessment ^4^. RAAB7 was launched as the methodology supported by ICEH in December 2021. The legacy software underlying RAAB5 and RAAB6 is no longer supported by its developers and cannot be maintained.

RAAB surveys are used to monitor vision impairment prevalence as an indicator of population eye health and effective coverage of cataract and refractive error services as indicators of service access and quality, typically at a subnational level. These data are primarily used to inform eye health planning and policy development ^5, 6^. To ensure consistency and quality, the RAAB methodology is delivered by a network of qualified Trainers who themselves have received standardised training in the sampling and examination protocols.

RAAB has a different purpose to bespoke comprehensive eye health surveys which provide more information than RAAB but are also more expensive. In contrast to comprehensive eye health surveys, RAAB is not designed to estimate the prevalence of specific eye conditions or provide estimates for population strata. Also, RAAB does not collect extensive sociodemographic and general health data or include ocular imaging, visual fields assessment or genetic sampling.

An important secondary use case for RAAB data has been its contribution to modelled estimates of global blindness. In the 2020 Global Burden of Disease (GBD) study by the Vision Loss Expert Group, RAAB data were 46% of all primary data sources for vision impairment prevalence estimates and 61% of all primary data sources for causes of vision impairment estimates ^7, 8^. In turn, the International Agency for the Prevention of Blindness’s Vision Atlas is populated by the outputs of the GBD study. RAAB data were also the majority of primary data sources for WHO’s 2022 baseline report on effective coverage of eye care services globally ^9^. In this way, RAAB data made available open access, are foundational to the headline numbers used in supranational advocacy for eye health.

The global eye health policy and advocacy landscape has changed since the inception of district-level rapid cataract blindness surveys in 1995. WHO’s 2019 World Report on Vision called for countries to work towards integrated, people-centred eye care, with eye health positioned as an integral component of countries’ universal health coverage (UHC) agenda and the 2030 Sustainable Development Goals ^10^. RAAB7’s core aims and objectives remain aligned with the rationale for the original survey design outlined by Dr Limburg and colleagues – to provide good-quality population data for eye care service planning and monitoring at a much lower cost than would be incurred using comprehensive survey methods. The RAAB7 project has provided an opportunity to reflect on what data the survey should collect and what outputs are most useful for its users as the eye health sector aims to meet important UHC targets set for 2030.

Two previous publications have outlined some of the early priorities of the RAAB7 project ^11, 12^. In this paper, we give an updated overview of RAAB, describe the survey methodology in 2023 and highlight priority areas for future research and development.

## The RAAB7 project

At its inception in 2018, the goals of the RAAB7 project were to fully digitise RAAB and to ensure the outputs were optimised for eye health planning. The project aimed to make data collection faster, more accurate and more secure while researching and developing modifications to the core methodology as well as new optional data collection modules and revising the automated reporting available to users. The project also aimed to rebuild the RAAB data repository as the end point of RAAB7’s digital data workflow, including a new front-end website ( https://www.raab.world) for improved access to the past 20 years of RAAB surveys worldwide.

### Ethics and governance

**Figure 1.**
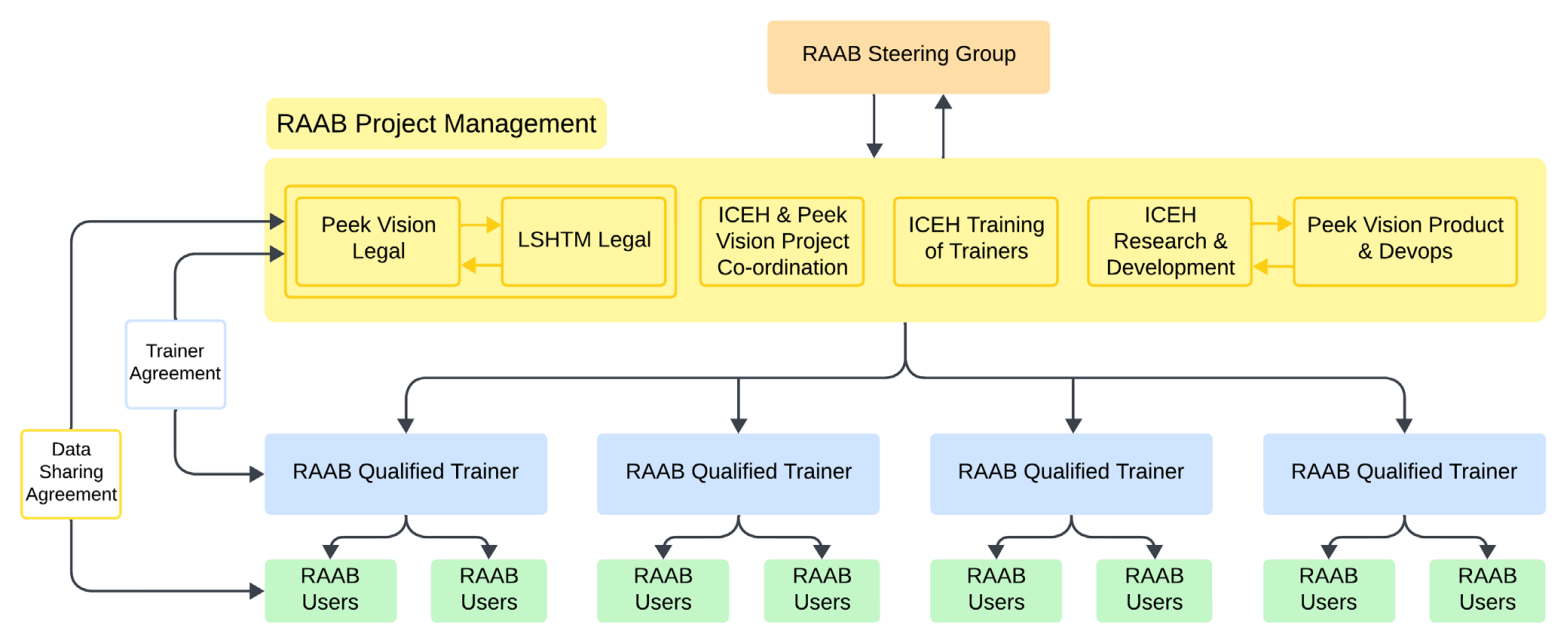
Relationships between RAAB stakeholders. This diagram is intended to convey the dissemination of RAAB methods from the project team to end users via a network of trainers. The number of active RAAB trainers is greater than four. RAAB users are teams implementing surveys.

Ethical approval for the RAAB7 project was granted by the London School of Hygiene & Tropical Medicine Ethics Committee (Ref 15504). RAAB7 has a project management team at ICEH which is overseen by an international Steering Group that consists of representatives from international non-governmental organisations ( https://www.raab.world/about-raab/who-we-are) ( Figure 1). For individual RAAB surveys, a nominated Principal Investigator is responsible for all local ethical approvals and permissions necessary to conduct the survey.

## RAAB7 sampling and examination protocol

### Study objectives

RAAB surveys are done to:

- Estimate the prevalence of blindness and vision impairment in the population aged 50 years and older
- Report the principal causes of blindness and vision impairment in the sample
- Estimate effective cataract surgical coverage (eCSC) and cataract surgical coverage (CSC) in the population aged 50 years and older
- Describe cataract surgical outcomes among all operated eyes in the sample
- Report main barriers to cataract surgery among people with unoperated cataract
- Estimate distance effective refractive error coverage (eREC) and refractive error coverage (REC) in the population aged 50 years and older

### Target population

Since inception, the population of interest for RAAB has been people aged 50 years and older ^1^. The prevalence of blindness is higher in this age group than younger age groups which allows for a smaller sample size–and therefore less resources–compared to doing an all-age survey. Estimates of blindness prevalence and causes among people 50 years and older were found to be a reasonable proxy for the total population in an analysis of the 1996 Gambia national eye health survey ^13^. We re-examined this rationale for sampling the population 50 years and older by comparing the data from 1996 with findings from the 2019 Gambia national eye health survey, which included people 35 years and older ^14, 15^. In 2019, the distribution of blindness prevalence by age group was comparable with 1996 results ( *Extended data*). People may be at risk of vision impairment from glaucoma and diabetic retinopathy from a younger age than from age-related cataract. This poses the question whether RAAB should survey the population 40 years and older. Surveying this age group would also give a more complete understanding of presbyopia and near effective refractive error coverage. Using the same 2019 Gambia survey data, we reviewed the implications of lowering the lower age limit for a hypothetical RAAB sample. Sampling a population 40 years and older instead of 50 years and older increased the sample size by almost 50%; this would take four data collection teams an extra three weeks of field work to complete the additional clusters required ( *Extended data*). Even without lowering the eligible age threshold, standard RAAB sample sizes will become larger and, therefore, costlier to survey as the prevalence of blindness in populations 50 years and older decreases globally in years to come. Therefore, the existing lower age limit of 50 years remains appropriate for fulfilling the methodology’s requirement to be rapid and relatively inexpensive.

### Sampling methodology

RAAB7 uses the two-stage cluster random sampling approach described by Kuper *et al.* in 2006 ^3^. At the first stage, census enumeration areas are selected as primary sampling units (PSU) with probability proportionate to their all-age population size at the time of the latest census. If a census is known to be significantly outdated or PSU population sizes are known to have increased or decreased at unequal rates across the sampling area, an alternative sampling frame can be made from alternative boundaries e.g., electoral wards. In an area undergoing significant inward or outward migration, it may be appropriate to delay a RAAB until the population is more stable.

To avoid the time and cost of developing household- or individual-level sampling frames per selected PSU, at the second stage, 50 individuals aged 50 years and older are enrolled using a sketch-mapping cluster segmentation approach. Boundary maps of census enumeration areas can be requested from national statistics offices or drawn with local guidance if these are unavailable. Community input is then required to segment the map according to population size. The estimated proportion of the all-age population that is aged 50 years and older is used to determine the required segment size to enrol a cluster of 50 people. For example, if 20% of the all-age population is estimated to be aged 50 years and older, the survey team should create segments of approximately 250 people of all ages. For a PSU of population 1000, we assume people aged 50 years and older are distributed evenly among the population, divide the PSU into four equal segments by population size (1000/250) and select one at random. A PSU with less that 500 people is surveyed without segmentation and enrolment continues in the nearest population unit if less than 50 people 50 years and older live there. Teams start from a randomly selected corner of a segment (i.e., north-east, south-east, south-west or north-west) and proceed systematically, door-to-door, to enrol participants. To minimise selection bias, eligible but unavailable participants are enrolled then followed up at the end of the day to be examined or finalised as unavailable.

To make the survey faster and more affordable, participant enumeration and examination are done on the same day. Based on experience of the typical time taken for travel, orientation and data collection, the number of participants per cluster is fixed at 50 to allow one team to complete one cluster per day. In areas with lengthy daily travel time to and from clusters a cluster size of 40 may be appropriate. Options for clusters of 35 or 60 are permitted if the optional Diabetic Retinopathy module (see below) is enabled. This predictable data collection schedule allows for simpler and more efficient survey logistics.

The overall probability of any person aged 50 years and older in the target population being selected is approximately equal (but unknown) and the sampling design is therefore approximately self-weighting. No survey design weights are applied during analysis, but the age group-gender profile of the sample is compared with the population age-gender structure from census data and a poststratification weighting is applied to sample estimates of prevalence and coverage (see below).

### Sample size calculation

The RAAB7 sample size calculator requires the inputs in Table 1.

**Table 1.** Inputs required for RAAB sample size calculator.

Term	Explanation and RAAB calculator options
P	Anticipated prevalence of blindness in the population 50 years and older
D	Relative precision of estimate (from 10% to 30% in 1% steps)
d	Anticipated non-response rate (from 0% to 20% in 1% steps)
DEFF	Design effect for cluster size (one of 1.4, 1.5 or 1.6)
Z (1.96)	Confidence level for interval (fixed at 95%)

The prevalence of blindness (P) is estimated from previous survey results in a sampling area, with consideration for the anticipated trend in blindness over time. Estimates from neighbouring settings with similar eye health system maturity are applied if none are available for the sampling area of interest. Where there is more than one estimate available it is prudent to use the lowest reasonable estimate to ensure the survey is not underpowered. By using the anticipated prevalence of blindness, surveys are adequately powered to estimate eCSC as the prevalence of blindness is expected to be lower than the prevalence of (operated and unoperated) cataract cases needed to estimate a sample size for eCSC. The confidence level for the uncertainty interval around the blindness estimate (Z) has been fixed to 95%, i.e., if we repeated a RAAB in the same population 100 times, we would expect 95 of the (different) intervals generated to include the true population prevalence of blindness. The median response rate among 291 surveys with values available on the RAAB repository (see below) in January 2024 was 96.8% (interquartile range 94.8% – 98.4%). This typically high response rate is likely because surveys are most often done in rural or mixed rural and urban areas in older populations in low- and middle-income countries where people tend to be at home or working near home. To err on the side of caution, the default non-response rate (d) remains 10% but can be set anywhere between 0–20% in one percentage point steps. To account for the effect of the cluster sampling approach on the statistical power of the sample size, a design effect (DEFF) is applied according to the selected cluster size. DEFF, and therefore the final sample size, increases with increasing cluster size. The calculator uses a DEFF of 1.4 for cluster sizes 35 and 40, 1.5 for cluster size 50 and 1.6 for cluster size 60. These values were chosen based on analysis of early RAAB surveys.

$$\text{S}=\left(\frac{Z^{2}P\left(1-P\right)}{D^{2}}\right)/\left(1-d\right)*DEFF$$

### Eligibility and exclusion criteria

Data collection teams move from household to household. All adults aged 50 years and older in the general population are eligible to be enrolled if they reside in the household (e.g. share meals from the same kitchen) at least 6 months per year. This definition of “resident” can be modified to suit a specific survey context if it is applied uniformly, but temporary visitors present on the day of data collection are excluded. Adults aged 50 years and older living in institutions are excluded as one home for the elderly may provide an entire cluster of participants who may not be representative of the general population aged 50 years and older.

Teams must ask about any eligible people at every household. If a household is empty or locked, they ask neighbours about the residents. Anyone who is eligible but unavailable, is enrolled in the survey – their name, age and gender are collected from family members or neighbours and added to a “mop up” list in the RAAB7 app. Everyone on the mop up list is revisited before the teams leave the cluster to attempt to examine them a second time. If they are still unavailable, their examination record is finalised as such. Survey planners should allow for the possibility of clusters with a low response rate to be revisited on additional days.

### Written informed consent

A template study protocol developed by ICEH is shared with all prospective RAAB7 users ( https://doi.org/10.17037/PUBS.00003744). This contains a participant information and written consent sheet that includes essential information for participants, including the fact that their anonymous data may be included in the online open access RAAB repository and may be included in secondary analyses. Information and consent sheets are translated into local languages by each RAAB user and given to, or read out to, participants before they enrol in the survey. Participants sign or thumbprint a paper copy of the consent form. The study protocol and informed consent process must be approved by a local ethics committee before a survey can begin.

### Distance visual acuity screening protocol

Distance visual acuity (VA) screening is aligned with the International Classification of Disease (ICD-11) vision impairment threshold categories ( Table 2) ^16^. Participants are screened down to 6/12 acuity level (i.e., normal vision), with other thresholds at 6/18, 6/60, 3/60, 1/60, light perception, and no perception of light. Impairment at the person level is based on presenting visual acuity (i.e., with correction if available) in the better eye. Acuities 1/60, light perception, and no perception of light are all classified as blindness.

**Table 2.** Thresholds for vision impairment categories in RAAB based on presenting visual acuity in the better eye.

Vision impairment category	Worse than (Snellen VA)	Better than or equal to (Snellen VA)
Normal	-	6/12
Mild	6/12	6/18
Moderate	6/18	6/60
Severe	60	3/60
Blind	3/60	-

Vision assessment is done using the Peek Acuity vision test, integrated into the RAAB7 data collection app. This digital version of the tumbling E chart presented on a mobile phone or tablet display screen has been validated against logMAR and Snellen vision tests in older adults ^17^. It presents single high contrast optotypes (black on a white device screen at full brightness) in a bounding box at a distance of 3 metres or 1 metre according to the threshold tested. Optotype size must be calibrated for each device before first use by measuring screen display and optotype width and height. Test distances are measured using a pre-cut and marked rope, held between examiner and participant. Starting at 6/60, participants must correctly identify 4 out of 5 optotypes to pass at a given threshold and proceed to the next. Participants indicate the direction of the open end of the “E” optotype and the examiner swipes the device screen in the same direction to mirror their response. The result is automatically passed to the data collection form and users can choose to see acuity levels presented in Snellen (metric or imperial), logMAR or decimal notation. The Peek Acuity test must be done indoors; it uses mobile devices’ in-built luxmeters to warn users about excessive ambient light that decreases the accuracy of the test. If indoor examination is expected to be a barrier to data collection, tumbling E charts printed on cardboard can be used outdoors and the results manually entered into the RAAB7 app.

**Figure 2.**
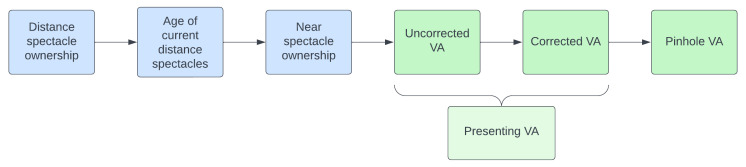
The spectacle ownership and distance visual acuity screening workflow in RAAB7.

The RAAB7 workflow for spectacle ownership and vision screening is shown in Figure 2. All participants are asked about their use of distance spectacles and have their uncorrected VA screened in each eye, i.e., right eye then left eye, at the thresholds outlined above. Distance vision is not screening with both eyes open simultaneously in RAAB. Participants who own and habitually use distance correction then put their distance spectacles on and have their corrected VA screened in right and left eyes. Presenting VA is a secondary variable, i.e., not directly measured in RAAB7. It is a composite measure of uncorrected VA and corrected VA; presenting VA is equal to uncorrected VA if the participant does not wear distance spectacle and is equal to corrected VA if they do wear distance spectacles ( Figure 2).

Pinhole VA is tested in any eye that has presenting VA worse than 6/12 but better than light perception. For eyes with presenting VA of light perception or no perception of light the pinhole VA value automatically defaults to match the presenting VA value, and the test is not presented. Pinhole VA may be tested in none, one or both right and left eyes per participant, according to the preceding presenting VA results and should be held in front of (outside) any distance spectacles as the prescription may not be optimum. Objective and/ or subjective refraction are not compatible with RAAB’s rapid examination protocol as they require additional equipment to be carried door-to-door and additional time to complete. Pinhole VA has been shown to be an adequate proxy for best-corrected VA from refraction in adults 50 years and older ^18^, but may under-estimate the number of people with refractive error (according to the definition set out below) ^19^. A multi-pinhole occluder with openings 0.5–1.0mm is recommended based on experience and user feedback.

UCVA was added to the VA screening workflow in 2021 in response to the call by WHO for the sector to report met need for refractive error correction alongside unmet need ^10, 20^. RAAB aligns with the ICD-11 recommendation to collect uncorrected, presenting and best-corrected visual acuity in epidemiological surveys. Measuring UCVA allows for the ‘gold standard’ effective refractive error coverage calculation to be reported ^21^. A question on the age of a participant’s current distance spectacles (less than 2 years, 2 to 5 years, more than 5 years) has been included in RAAB7 to elucidate whether undermet need for refractive error correction (or the ‘quality gap’) is due to recent but inappropriate spectacles, a service quality issue, or, whether older spectacles might indicate that access to services is an issue for the participant to obtain more up-to-date correction ^22^.

### Ophthalmic examination protocol

Examiners conduct a lens examination for both eyes of every participant using pen torch and distant direct ophthalmoscopy, regardless of their distance visual acuity. This is necessary to capture information on successful cataract surgery. One of seven lens status options is selected ( *Extended data*).

An eye with 6/12 presenting VA is categorised as normal, with no cause assigned, even if eye disease e.g., early cataract is present.

For an eye with distance presenting VA worse than 6/12, RAAB examiners assign a main cause of vision impairment, and then a principal cause of vision impairment in the person, according to a defined list of eye conditions ( *Extended data*). An eye that improves from worse than 6/12 (uncorrected or corrected) to 6/12 with pinhole is assigned refractive error as the main cause, even in the presence of other eye disease. Otherwise, if two conditions are observed in the same eye, examiners determine if one cause is secondary to the other and record the primary cause. If this is not possible, they assign the cause more responsible for vision loss. For example, in an eye with cataract and suspect glaucoma, if cataract surgery would be unlikely to improve vision due to advanced optic disc damage, they would assign glaucoma as the main cause.

Any eye with pinhole VA worse than 6/12 not due to cataract or corneal opacity or an obvious globe abnormality is dilated to allow for a more detailed examination of the posterior segment.

When assigning the principal cause of vision impairment in the person, examiners consider the main cause in both eyes. The main causes in right and left eyes are listed in a specific order, with causes more easily treatable or preventable towards the top. This approach of ranking eye conditions is derived from the 1988 WHO Eye Examination Record for blindness surveys ^23^.

If each eye has a different main cause, the cause which is more readily treatable or preventable (i.e., higher up the list) is selected as the principal cause in the person. In this way, causes of avoidable vision impairment (more likely to be controlled by public health strategies) are prioritised.

RAAB users can opt to remove an eye condition that is not endemic in their setting (e.g., trachoma or onchocerciasis) and/or add pterygium or myopic macular degeneration if they are common causes of vision impairment. Previously, the total number of conditions listed was fixed at 13 and substitution was required but for RAAB7 the list of causes can be expanded as relevant. In 2024, anterior uveitis and other (non-cataract) surgical complications will be made available as optional causes, based on user feedback.

### Cataract follow up questions

A series of follow up questions are answered for any participant with a history of cataract surgery, including an examiner assessment of the reason for post-operative presenting VA worse than 6/12. Participants with obvious lens opacity and PinVA <6/12 are asked why they have not had cataract surgery. Examiners choose up to two response options from a list of six ( *Extended data*).

### Optional data collection

Two optional user-defined questions can be included in the standard RAAB questionnaire. These are typically used to collect sociodemographic data such as education level, ethnicity, etc. Trainers define the question and any number of response options in the RAAB7 software. RAAB users should have an analysis plan for these data before including them as they cannot be integrated into the automated RAAB7 report (see below).

Two optional data collection modules are currently available in RAAB7: a Diabetic Retinopathy module (DR module) and a Disability module. When these modules are included, additional fields are presented in the RAAB7 app after the core RAAB questionnaire.

The DR module has two components. Firstly, an assessment of the diabetes status of all participants, including known history of diabetes, time since last diabetic eye examination and a random blood glucose test ( *Extended data*). Secondly an assessment of retinopathy among known or suspected diabetics. Retinopathy and maculopathy are graded according to the Scottish Diabetic Retinopathy Grading Scheme ( *Extended data*) ^24, 25^. The expanded protocol for the DR module requires an additional team member and examination equipment (see below) and smaller cluster sizes–and therefore larger DEFF, to accommodate the longer examination times. Team training (see below) is one day longer and includes a fundus photo grading interobserver variation (IOV) exercise for ophthalmologists. The anticipated prevalence of diabetic retinopathy should be used for P in the sample size calculation if it is predicted to be lower than the anticipated prevalence of blindness.

The Disability module uses the Washington Group Short Set (WG-SS) on Functioning to record self-reported functional limitations in any of six domains: seeing, hearing, mobility, cognition, self-care and communication ^26, 27^. The option to use the Short Set – Enhanced (WG-SS Enhanced), which includes additional questions on upper body functioning and anxiety and depression, will be available in 2024 ( *Extended data*) ^28^. The Washington Group questions do not require any additional resources, but data collectors must be trained to administer the questions appropriately and in a standardised way.

Referral pathways for participants identified with eye conditions requiring treatment must be agreed with local service providers in advance of data collection, in line with local policy. Similarly, guidance must be available for any participant identified with raised random blood glucose or functional limitations in the DR and Disability modules respectively.

### RAAB protocol limitations

To save time and resources, RAAB’s second stage sampling does not include individual level listing or compact segment sampling of clusters as described by Turner *et al.* ^29^. In this way, all individuals in the target population do not have a known non-zero chance of selection; however, eligible people not enrolled in any segment containing more than 50 people aged 50 years and older are not expected to be systematically different from those enrolled. The risk of selection bias is expected to be lower than if a random walk method without full enrolment of non-responders was used.

Sampling the population 50 years and older is essential for the rapid methodology. However, a reliance on RAAB data in eye health planning may inadvertently deemphasise the importance of estimating the need for eye health services among younger age groups, particularly for refractive error and non-vision-impairing primary eye care. Other sources of population data should be considered for a complete picture of population eye health need.

RAAB does not measure participants’ full visual acuity and does not screen any lower than 6/12. This screening approach has been shown to have good sensitivity and specificity to detect blindness, severe vision impairment and moderate vision impairment compared to a comprehensive vision assessment ^30^. The protocol assigns a principal cause of vision impairment per participant in such a way that multimorbidity is not captured. The emphasis on quantifying avoidable (treatable or preventable) causes of vision loss that are most amenable to public health interventions is important for planning to control vision impairment but is likely to over-estimate the contribution of anterior segment conditions (particularly refractive error and cataract) while under-estimating posterior segment conditions contributions to vision impairment ^30^. For example, RAAB under-estimates the magnitude of glaucoma as eyes with 6/12 vision are categorised as normal, even in the presence of advanced glaucomatous visual field loss. A bespoke, comprehensive survey design is required to estimate the prevalence of specific eye conditions in a population.

Where the DR Module is done, RAAB’s same day enumeration and examination do not permit fasting blood glucose tests. The random blood glucose test may over-estimate the prevalence of suspected (i.e., not previously diagnosed) diabetes.

## RAAB survey personnel and equipment

### Data collection teams

A standard data collection team includes an ophthalmologist and a non-medical eye health worker such as an ophthalmic nurse, ophthalmic clinical officer or refractionist. Each team requires a driver and vehicle. A team of cluster informers works in parallel to the data collection team to sensitise people within clusters in advance of the data collection. Cluster informers may be community health workers or similar. They may be engaged to do the sketch-mapping and cluster segmentation and can organise a local guide to support data collectors with orientation in the cluster and any translation required. A RAAB survey with the DR module requires an additional team member–typically a general nurse, to measure random blood glucose.

The number of data collection teams used depends on available resources. More teams will complete data collection more quickly; however, more than six teams makes it difficult to ensure consistency in adherence to the protocol throughout data collection and to assess consistency via the training week IOV exercise (see below) and is not recommended for these reasons.

### Ophthalmic equipment

Each data collection team should carry the following equipment for a standard RAAB examination: Android mobile device for data entry and VA screening, rope to measure 3m and 1m for visual acuity testing, pen torch, direct ophthalmoscope, dilating eye drops (and optional tumbling E chart cards for outdoor use). Additional clinical equipment is required if doing the DR module: indirect ophthalmoscope, digital glucometer and test strips, alcohol swabs, single-use safety lancets, disposable gloves, cotton wool, sharps disposal box.

## Trainers and training week

### RAAB Trainers

The RAAB survey methodology is applied consistently via a network of qualified RAAB Trainers. A RAAB7 qualified Trainer has themselves received standardised training and assessment from ICEH in the sampling method, examination protocol and use of the software for data monitoring and report generation.

Training of Trainers was previously done by Dr Limburg face-to-face as a one-week intensive course in Kenya in 2007, Panama in 2012 and Philippines and UK, both in 2014. In late 2023, a new cohort of prospective trainers completed the first online version of this course. Trainees must lead a training week under supervision by a senior Trainer as part of a structured observation assessment before being added to the pool of available qualified Trainers.

RAAB7 qualified Trainers sign an agreement with ICEH that outlines the roles and responsibilities of both parties and confirms they will adhere to best practice and data sharing and privacy obligations. Trainers are independent of ICEH and the RAAB7 project and define their own terms of reference and remuneration (per diems, travel, accommodation, etc.) with RAAB users. The RAAB repository website has a “trainer area” accessible to trainers only which provides standardised resources from ICEH for training data collection teams and updates on software functionality, as well as survey support documents and templates.

### RAAB Training Week

Just before a RAAB starts, Trainers travel to the site of the survey to conduct a face-to-face training week with data collection teams. Training week includes an IOV exercise to ensure comparability of examination findings across teams. Agreement on a subset of variables (VA, lens status and cause of vision impairment) is graded using the kappa statistic, with a value ≥0.6 considered acceptable agreement. A pilot cluster is examined on the final day of the training week. All teams attend the same cluster and enrol 50 participants between them.

## RAAB user agreement

Before starting data collection, a RAAB user (i.e. the organisation owning the data and implementing or facilitating a survey) signs a tri-party agreement with LSHTM and Peek Vision setting out the roles and responsibilities of each party. This includes responsibilities of the RAAB user to gain local ethics approval and ensure that written informed consent is sought from survey participants. RAAB survey data are owned by RAAB users. The agreement specifies how survey data are stored and processed on behalf of the user by ICEH and Peek Vision. It also provides users with legal assurance that both parties will comply with data protection laws including the European Union and United Kingdom’s General Data Protection Regulation (GDPR). The agreement gives the data owner options to specify the extent of open access data sharing they agree to on the repository following an 18-month embargo period. The metadata for all completed RAABs are sent to the repository. Thereafter, data owners can elect to also share their main results (vision impairment prevalence, effective coverage, etc.), main results and RAAB7 report, or their main results, report and the participant level dataset. Previously, some RAAB5 and RAAB6 data held only locally have been lost due to loss or damage of computer hardware. The user agreement also stipulates that a copy of the dataset is by default transferred to a secure server accessible only on site at LSHTM for safekeeping. Data are not retained on the Peek Vision Amazon Web Services (AWS) platform long-term, therefore, LSHTM offers this service as a means of protecting RAAB data from accidental loss. The secure data server is accessible only to registered members of the ICEH project team and is supported by automatic daily back up and file access auditing.

Currently, the majority of the costs incurred by ICEH and Peek Vision for the ongoing development and delivery of the survey methodology and software and the survey trainer support services are met through grants. In addition, as part of the agreement to receive access to the software and support services, the user organisation pays a fee to Peek Vision and ICEH, to contribute towards to cost of providing the tool and associated services.

## RAAB7 software and data flow

### Software and data flow

The RAAB7 software has two components: a web browser-based “Admin” platform for RAAB trainers and coordinators to set up and manage data collection, and a custom-built application (app) for data collection on Android mobile devices. Data can be collected offline but are automatically synchronised to an AWS cloud-based server via a secure encrypted connection when mobile devices are online. In this way, trainers and coordinators can monitor data collection in real time (as connectivity permits). The app uses in-built validation logic to guide data collectors towards more likely responses, based on information entered in preceding questions. For example, if presenting VA is less than 6/12 and pinhole VA is 6/12 in an eye, refractive error will be highlighted among the list of causes of vision loss. The app workflow is designed to minimise data entry errors in the field; any remaining examination protocol violations are flagged in Admin for review by the survey management team.

**Figure 3.**
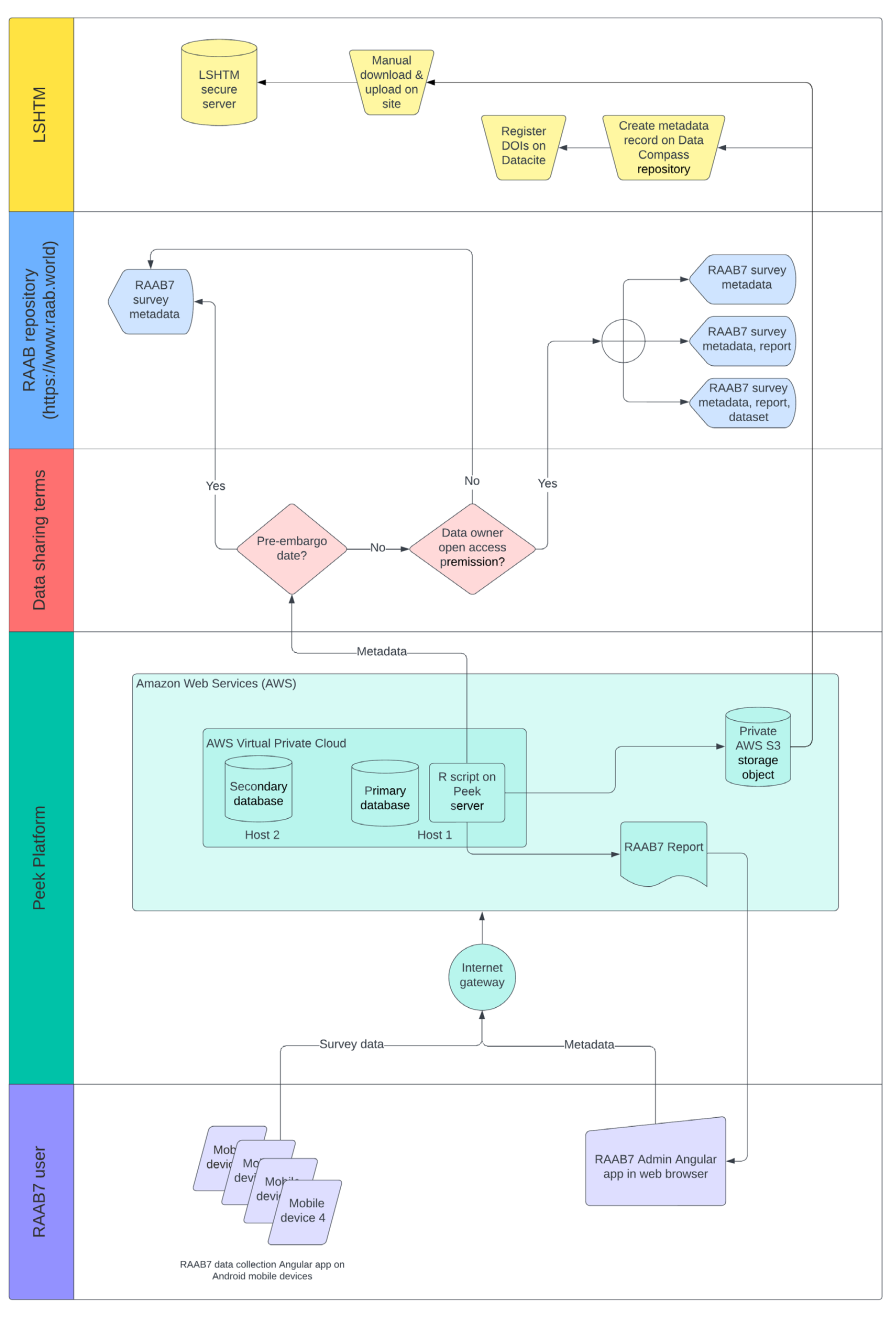
Diagram showing data flow through the RAAB7 project.

On completion of fieldwork and any data cleaning for flagged examination records, duplicate participant IDs etc., the trainer agrees the final version of the dataset with the PI, “closes” it–meaning no further modifications are possible on the Admin platform, and generates an automated report (see below). The process of creating a report automatically exports a metadata record for the completed survey from the Peek Vision platform to the RAAB data repository (see below) ( Figure 3). Survey metadata inputted on the Admin platform includes the year of completion, geographic location of the survey, including cartographic information from OpenStreetMap ( https://www.openstreetmap.org/), the PI and trainer names, the sample size and response rate, and whether or not the DR module and/ or Disability module were used. It also includes the end date of the 18-month embargo period.

When a RAAB survey embargo period elapses, the survey can be re-exported from the Peek platform to the RAAB repository. At this time, the report, or the report and the participant level dataset, will also be made available on the repository along with the metadata.

### Data security

The RAAB7 software is developed and hosted by Peek Vision on an AWS Virtual Private Cloud. Peek Vision is an ISO 27001 certified organisation meaning it complies with the foremost international standard for information security management systems ^31^. RAAB is hosted in two AWS Regions–one in the European Union (Frankfurt, Germany) and one in Mumbai, India; users can choose which to host in based on the data protection and security requirements of their jurisdiction. Each individual RAAB dataset is separated and accessible only via authorised user log-in. The RAAB7 app runs on Android mobile devices (version 8 or later). It is password-protected and enforces full disk encryption and a six-digit device PIN. Further information is available on the RAAB website ( https://www.raab.world/about-raab/about-raab-data/data-security-and-protection).

## Data analysis and RAAB repository

### RAAB7 analysis and reporting

RAAB has always provided automated, standardised epidemiological reporting, available to users immediately upon completion of a survey. Users do not need to employ additional personnel to do bespoke data analysis. In consultation with the Steering Group, we developed a RAAB7 report that consolidates priority outputs from over 100 tables and figures generated in RAAB6 into a single pdf report. The RAAB7 report includes new calculations for key eye health indicators including effective cataract surgical coverage and effective refractive error coverage ^22, 32^ and adds additional narrative context and definitions to support interpretation.

Surveys are not powered for subgroup analyses; however, the report does disaggregate all output by male and female gender. A few key outputs are disaggregated by disability status (any disability, any non-vision related disability, no disability) when the Disability module is done. In future, outputs disaggregated by other equity dimensions such as socioeconomic position (see below) are likely to be restricted to two or three groups given the relatively small sample sizes typically surveyed.

Standard errors for 95% confidence intervals are calculated to account for the clustered survey design according to formula 6 in Bennett *et al.* ^33^ Age-gender weighted prevalence and coverage estimates are post-stratified using population age-gender counts from census data. Post-stratification weights crude numbers of cases by 10-year age group and gender groups in the sample (male and female for each of the 50–59, 60–69, 70–79, and ≥80 year age groups) to the corresponding known distribution of the target population and is beneficial when certain age-gender groups are over- or under-sampled. In RAAB, it is not uncommon that younger age groups are under-sampled and older age groups over-sampled due to older people being more likely to be at home, a scenario that can inflate crude vision impairment prevalence.

The code for RAAB7 analysis is written in the R open source statistical computing software (R Foundation for Statistic Computing, Vienna, Austria) and is available at https://github.com/raabteam/raab7-analysis.

### Data repository

A repository of RAAB metadata (i.e., “data about data”) and individual participant level survey datasets was first made publicly accessible via a front-end website in 2013; users could download reports and datasets where owners had agreed to make them open access. The process of requesting permission from PIs that their survey data were shared online was retrospective and often unsuccessful.

In 2021, ICEH took over management of the RAAB repository from Dr Limburg and developed a new website for public access to RAAB outputs, available at https://www.raab.world. RAAB7 has been designed to support open data in eye health by defining data sharing permission with users in advance (see RAAB user agreement section above). Where agreed, the centralised platform allows the RAAB team to store data and host it on the repository following the embargo period without having to seek out data and written permission as a separate administrative process.

We have developed the new repository to align with the FAIR principles for data management and stewardship, which stipulate that all research output should be Findable, Accessible, Interoperable and Reusable ^34^. Each RAAB dataset and report is described using Dublin Core Metadata Initiative (DCMI) elements ^35^ and has been assigned a Digital Object Identifier (DOI) via the LSHTM Library and these will be accessible on the repository in 2024. The webpages for each dataset and report will include human- and machine-readable metadata. Repository users are required to register for a free user account to download data or reports. Registration is via a short form accessible on the website. We believe this step is justified to allow us to understand what type of user is accessing RAAB data (e.g., Ministry of Health, NGO, academic, student, etc.) and for what purpose so we can continue to develop the repository to best meet the eye health community’s needs.

Recommended citations for datasets and reports are included in their metadata. We encourage anyone using RAAB survey datasets in a secondary analysis to list the “RAAB International Co-author Group” as a group (corporate) author. This group includes PIs as representatives of primary data collection teams if they acknowledge they have read the manuscript and consent to be named as a collaborator, as per International Committee of Medical Journal Editors (ICMJE) guidelines. Collaborators are individually cited in MEDLINE/PubMed where the Group author name is included in an article byline.

In parallel, a renewed effort has been made to contact historic RAAB PIs to request they make data available open access via a Data Transfer Agreement with LSHTM. Some datasets available on the original repository were removed pending new agreements while many others have been made available for the first time. Historic RAAB datasets are processed to align with RAAB7 variable names and values as they become available, so they are compatible with the open access code developed for RAAB7. We have created RAAB7 format reports for all available datasets to improve comparability with new surveys. Reports and csv data files in the RAAB7 format are available for download to registered users.

As of January 2024, the repository lists 375 surveys done since 2000 with 164 (44%) having open access data and reports ( Table 3).

**Table 3.** Proportion of RAAB surveys with open access data per Global Burden of Disease super region.

GBD Super region	Available		Unavailable		Total	
	N	%	N	%	N	%
Central Europe, Eastern Europe & Central Asia	6	75.0	2	25.0	8	100.0
High Income	4	100.0	0	0.0	4	100.0
Latin America & Caribbean	17	81.0	4	19.0	21	100.0
North Africa & Middle East	8	30.8	18	69.2	26	100.0
Southeast Asia, East Asia & Oceania	67	51.9	62	48.1	129	100.0
South Asia	27	25.5	79	74.5	106	100.0
Sub Saharan Africa	35	43.2	46	56.8	81	100.0
**Total**	164	43.7	211	56.3	375	100.0

## Active research and development

### Near vision screening

In 2021, Peek Vision validated a mobile app-based near VA screening test that uses the same tumbling E test design as RAAB7’s existing Peek Vision distance acuity test ^36^. In 2024, this near vision test will be validated among people aged 50 years and older in a real world survey setting. The feasibility, i.e., time-cost, of screening all participants with better than light perception distance VA at a single near VA threshold, N6 at 40cm binocularly, will be investigated. If found to be feasible, this additional VA measure will be integrated into the core RAAB questionnaire and will allow RAAB7 to report near vision impairment prevalence and near vision eREC among the age group most affected by presbyopia. This will be an important development that contributes to addressing a significant data gap ^7^. Some additional questions on near glasses ownership and barriers to ownership are also being pilot tested and will be available along with near VA screening.

### Health systems module

Development of a RAAB Planning Module to support better use of survey data began in 2018 ^11^. This module has subsequently been reconceptualised as a standardised Health Systems data collection module to collect information from all eye health care facilities within a RAAB sampling area. Following a literature review and stakeholder interviews in 2022 (unpublished), indicators appropriate for subnational data collection have been selected from existing national eye health indicator lists ^37, 38^, with an emphasis on cataract, refractive error and diabetic retinopathy services. The pilot phase is focussed on identifying barriers and enablers to facility-level data collection in a variety of settings.

### Health economics module

A recent systematic review on the economics of eye health found data lacking in low- and middle-income countries ^39^. A RAAB Health Economics module is under development to collect data on the employment status and health-related quality of life of people with and without vision impairment, the affordability of eye care services and the extent to which there financial protection for people accessing services. In 2024, questionnaires will be pilot tested, first in a clinical setting, then within a RAAB7 survey.

### Monitoring eye health equity

In addition to existing gender and (optional) disability status options, we propose to include one or more socioeconomic position (SEP) indicators within the core RAAB questionnaire ^40^. We are testing the use of subjective SEP indicators to assess whether these quick-to-administer questions reliably demonstrate a socioeconomic gradient in eye health outcomes in RAAB surveys, as asset wealth questionnaires add additional time-cost to data collection and may not prove sufficiently discriminatory in subnational sampling areas.

## Future considerations

The research and development agenda outlined here was developed by the project management team in consultation with the steering group. For the next iteration of the research agenda, we will seek a broader range of RAAB stakeholder feedback to inform and prioritise research sub-projects. Online consensus and prioritisation projects have previously been used successfully in eye health agenda-setting and could be applied to RAAB ^41^.

### Other optional modules

Other modules proposed for future research and development include primary eye care and glaucoma. In some settings, non-vision impairing eye conditions can contribute considerably to the workload in secondary level facilities so quantifying the magnitude of all eye care need in a population will permit improved service planning.

RAAB under-estimates the magnitude of glaucoma as eyes with 6/12 vision are categorised as normal, even in the presence of glaucoma. As improved mobile tools for glaucoma detection become available, we aim to develop an extended protocol for a glaucoma module, similar to that used for diabetic retinopathy.

### Effective coverage monitoring

The emphasis on effective coverage indicators for eye health (eCSC and eREC) since the 2021 World Health Assembly endorsed targets for 2030 have prompted interest in using the RAAB platform to collect nationally representative data to allow countries to monitor service coverage semi-regularly. In 2024–2025, we will explore the viability of developing a “RAAB-lite” methodology, with a smaller sample size, lower age limit and narrower question set focussing on visual acuity, spectacle ownership and cataract operated/unoperated eyes.

### Sampling methodology

Over time, RAAB samples may become prohibitively large and costly to survey as blindness prevalence is reduced by ongoing development and public health interventions for eye care. To match RAAB sample sizes with available resources, the desired relative precision of the estimate may be decreased. Another option is to power RAAB sample sizes based on the prevalence of severe vision impairment or worse which will be higher than the prevalence of blindness. Any methodology update recommending this will be put to the RAAB steering group, should increasing sample sizes become problematic for a rapid survey approach; however, in the meantime, trainers will continue to use the prevalence of blindness as the default value for the calculator. Similarly, we will review the DEFF for blindness prevalence from historic and recent surveys by regions to determine whether or not the existing values in the sample size calculator remain appropriate.

Survey sampling relies on a recent census to develop an accurate sampling frame of primary sampling units. Where a census is known to be out of date, there is uncertainty in how representative the selection of PSUs with probability proportionate to size will be. In recent years, modelled gridded population estimates have been used to try to compensate for this, particularly for fast-growing urban populations in low- and middle-income settings ^42^. Cells in a gridded population dataset can be aggregated to clusters of the required population size and selected in lieu of census enumeration areas. We will review the age of census data used for sampling frames in RAAB7 surveys and investigate the potential utility of gridded population sampling for RAAB when resources permit.

### Examination protocol

Considering changes in the epidemiology of eye disease and treatment options over time, we will review the ordered list of causes of vision impiarment to determine if the existing contents and ranking are still appropriate.

As mobile technology improves in quality, usability and affordability, we will continue to review options for integrating ocular imaging into RAAB. Further integration with artificial intelligence (AI) -supported image reading could permit additional information to be reported from RAAB surveys. Large-scale collection of ocular images from low- and middle-income country surveys worldwide could help address the bias towards high-income country representation in the data AI models are trained on, if quality image capture is feasible and data sovereignty and security issues can be navigated ^43^.

### Global coverage, data gaps and accessibility

The World Report on Vision and the *Lancet Global Health* Commission on Global Eye Health both called for increased country coverage of population-based vision impairment studies and effort to address data gaps in certain regions ^10, 44^. Thus far, we have translated the RAAB7 software and supporting documentation into French and Spanish and will continue to build as diverse a library of translated documentation as resources permit. The 2023 cohort of trainee RAAB trainers included several Francophone participants to supplement the few existing ones, and we hope to recruit more Spanish-speaking trainers for the next iteration of the course. High-income countries are a significant data gap, with only a handful of RAAB surveys to date. To encourage uptake in future, we will consider what modifications to the methodology might be feasible to address lower response rates and likely reluctance to be examined “at the doorstep” in these settings.

## Conclusion

RAAB7 is a multi-year project to improve population-based eye health data collection, curation and analysis methods. The research and development agenda described above is not static and RAAB will continue to evolve to meet users’ needs. We wish to expand the use of RAAB to address data gaps worldwide and support eye health within universal health coverage.

## Data Availability

All open access RAAB datasets are available to registered users at https://www.raab.world. Access to The Gambia 2019 National Eye Health survey dataset was granted by Gambia study Principal Investigator and co-author (MJB) for the purposes of reviewing RAAB’s sampling strategy in this article. The Gambia 2019 dataset is not yet publicly available online while a series of publications are concluded. A link to the dataset will be added as soon as it becomes available. Open Science Framework: The Rapid Assessment of Avoidable Blindness survey: Review of the methodology and protocol for the seventh version (RAAB7), https://doi.org/10.17605/OSF.IO/UWD2E ^45^. This project contains the following extended data: Target population RAAB7 questionnaire Ophthalmic examination variables, response options and definitions Cataract follow up questions Diabetic Retinopathy (DR) module additional information References Data are available under the terms of the Creative Commons Attribution 4.0 International license (CC-BY 4.0).

## References

[1] Limburg H Kumar R Indrayan A : Rapid assessment of prevalence of cataract blindness at district level. *Int J Epidemiol.* 1997;26(5):1049–54. 10.1093/ije/26.5.1049 9363527

[2] Limburg H : Rapid assessment of cataract surgical services.2001. Reference Source

[3] Kuper H Polack S Limburg H : Rapid assessment of avoidable blindness. *Community Eye Health.* 2006;19(60):68–9. 17515970 PMC1871676

[4] Morjaria P Massie J Bastawrous A : A School Eye Health Rapid Assessment (SEHRA) planning tool: Module to survey the magnitude and nature of local needs. *BMC Public Health.* 2022;22(1): 1665. 10.1186/s12889-022-13927-x 36056322 PMC9437397

[5] Ramke J Zwi AB Silva JC : Evidence for national universal eye health plans. *Bull World Health Organ.* 2018;96(10):695–704. 10.2471/BLT.18.213686 30455517 PMC6238994

[6] Mathenge WC Hillgrove T Gisagara E : The Rwanda National Blindness Survey: Trends and use of the evidence to change practice and policy. *Afr Vis Eye Health.* 2021;80(1): a576. 10.4102/aveh.v80i1.576

[7] GBD 2019 Blindness and Vision Impairment Collaborators: Trends in prevalence of blindness and distance and near vision impairment over 30 years: an analysis for the Global Burden of Disease Study. *Lancet Glob Health.* 2021;9(2):e130–43. 10.1016/S2214-109X(20)30425-3 33275950 PMC7820390

[8] GBD 2019 Blindness and Vision Impairment Collaborators: Causes of blindness and vision impairment in 2020 and trends over 30 years, and prevalence of avoidable blindness in relation to VISION 2020: the Right to Sight: an analysis for the Global Burden of Disease Study. *Lancet Glob Health.* 2021;9(2):e144–60. 10.1016/S2214-109X(20)30489-7 33275949 PMC7820391

[9] World Health Organization: WHO Report of the 2030 targets on effective coverage of eyecare. Geneva,2022. Reference Source

[10] World Health Organization: World report on vision. Geneva,2019. Reference Source

[11] Mactaggart I Wallace S Ramke J : Rapid assessment of avoidable blindness for health service planning. *Bull World Health Organ.* 2018;96(10):726–728. 10.2471/BLT.18.217794 30455521 PMC6239001

[12] Mactaggart I Limburg H Bastawrous A : Rapid Assessment of Avoidable Blindness: looking back, looking forward. *Br J Ophthalmol.* 2019;103(11):1549–52. 10.1136/bjophthalmol-2019-314015 31266777 PMC6855783

[13] Dineen B Foster A Faal H : A proposed rapid methodology to assess the prevalence and causes of blindness and visual impairment. *Ophthalmic Epidemiol.* 2006;13(1):31–4. 10.1080/09286580500473787 16510344

[14] Hydara A Bastawrous A Bell S : The Gambia National Eye Health Survey 2019: survey protocol [version 2; peer review: 2 approved]. *Wellcome Open Res.* 2021;6:10. 10.12688/wellcomeopenres.16531.2 34796273 PMC8591516

[15] Hydara A Mactaggart I Bell SJ : Prevalence of blindness and distance vision impairment in the Gambia across three decades of eye health programming. *Br J Ophthalmol.* 2023;107(6):876–882. 10.1136/bjophthalmol-2021-320008 34949578 PMC10314069

[16] World Health Organization: ICD-11 for Mortality and Morbidity Statistics: 9D90 Vision impairment including blindness.2023; [cited 2024 Jan 18].

[17] Bastawrous A Rono HK Livingstone IAT : Development and Validation of a Smartphone-Based Visual Acuity Test (Peek Acuity) for Clinical Practice and Community-Based Fieldwork. *JAMA Ophthalmol.* 2015;133(8):930–7. 10.1001/jamaophthalmol.2015.1468 26022921 PMC5321502

[18] Kumar RS Rackenchath MV Sathidevi AV : Accuracy of pinhole visual acuity at an urban Indian hospital. *Eye (Lond).* 2019;33(2):335–7. 10.1038/s41433-018-0237-6 30341426 PMC6367369

[19] Loewenstein JI Palmberg PF Connett JE : Effectiveness of a pinhole method for visual acuity screening. *Arch Ophthalmol.* 1985;103(2):222–3. 10.1001/archopht.1985.01050020074024 3977693

[20] Keel S Cieza A : Rising to the challenge: estimates of the magnitude and causes of vision impairment and blindness. *Lancet Glob Health.* 2021;9(2):e100–1. 10.1016/S2214-109X(21)00008-5 33482137 PMC7816084

[21] Keel S Müller A Block S : Keeping an eye on eye care: monitoring progress towards effective coverage. *Lancet Glob Health.* 2021;9(10):e1460–e1464. 10.1016/S2214-109X(21)00212-6 34237266 PMC8440222

[22] McCormick I Mactaggart I Bastawrous A : Effective refractive error coverage: an eye health indicator to measure progress towards universal health coverage. *Ophthalmic Physiol Opt.* 2020;40(1):1–5. 10.1111/opo.12662 31879992 PMC7004023

[23] World Health Organization: Coding Instructions for the WHO/PBL Eye Examination Record (Version III).1988; [cited 2024 Jan 9]. Reference Source

[24] NHS Scotland: Scottish Diabetic Retinopathy Grading Scheme v1.1.2007; [cited 2024 Jan 17]. Reference Source

[25] Zachariah S Wykes W Yorston D : Grading diabetic retinopathy (DR) using the Scottish grading protocol. *Community Eye Health.* 2015;28(92):72–3. 27418727 PMC4944099

[26] Groce NE Mont D : Counting disability: emerging consensus on the Washington Group questionnaire. *Lancet Glob Health.* 2017;5(7):e649–50. 10.1016/S2214-109X(17)30207-3 28619216

[27] The Washington Group on Disability Statistics: WG Short Set on Functioning (WG-SS).2022; [cited 2024 Jan 4]. Reference Source

[28] The Washington Group on Disability Statistics: WG Short Set on Functioning – Enhanced (WG-SS Enhanced).2022; [cited 2024 Jan 4]. Reference Source

[29] Turner AG Magnani RJ Shuaib M : A not quite as quick but much cleaner alternative to the Expanded Programme on Immunization (EPI) Cluster Survey design. *Int J Epidemiol.* 1996;25(1):198–203. 10.1093/ije/25.1.198 8666490

[30] Zhang XJ Leung CKS Li EY : Diagnostic Accuracy of Rapid Assessment of Avoidable Blindness: A Population-based Assessment. *Am J Ophthalmol.* 2020;213:235–43. 10.1016/j.ajo.2019.12.009 31846622

[31] ISO: ISO/IEC 27001: 2022 Information security, cybersecurity and privacy protection.2022. Reference Source

[32] McCormick I Butcher R Evans JR : Effective cataract surgical coverage in adults aged 50 years and older: estimates from population-based surveys in 55 countries. *Lancet Glob Health.* 2022;10(12):e1744–e1753. 10.1016/S2214-109X(22)00419-3 36240806 PMC7618287

[33] Bennett S Woods T Liyanage WM : A simplified general method for cluster-sample surveys of health in developing countries. *World Health Stat Q.* 1991;44(3):98–106. 1949887

[34] Wilkinson MD Dumontier M Aalbersberg IjJ : The FAIR Guiding Principles for scientific data management and stewardship. *Sci Data.* 2016;3(1): 160018. 10.1038/sdata.2016.18 26978244 PMC4792175

[35] Dublin Core Metadata Initiative (DCMI). Reference Source

[36] Katibeh M Sanyam SD Watts E : Development and Validation of a Digital (Peek) Near Visual Acuity Test for Clinical Practice, Community-Based Survey, and Research. *Transl Vis Sci Technol.* 2022;11(12):18. 10.1167/tvst.11.12.18 36583912 PMC9807182

[37] McCormick I Mactaggart I Resnikoff S : Eye health indicators for universal health coverage: results of a global expert prioritisation process. *Br J Ophthalmol.* 2022;106(7):893–901. 10.1136/bjophthalmol-2020-318481 33712481 PMC9234411

[38] World Health Organization: Eye care indicator menu (ECIM): a tool for monitoring strategies and actions for eye care provision.Geneva: World Health Organization;2022. Reference Source

[39] Marques AP Ramke J Cairns J : The economics of vision impairment and its leading causes: A systematic review. *EClinicalMedicine.* 2022;46: 101354. 10.1016/j.eclinm.2022.101354 35340626 PMC8943414

[40] McCormick I Kim MJ Hydara A : Socioeconomic position and eye health outcomes: identifying inequality in rapid population-based surveys. *BMJ Open.* 2023;13(3): e069325. 10.1136/bmjopen-2022-069325 36882236 PMC10008479

[41] Ramke J Evans JR Habtamu E : Grand Challenges in global eye health: a global prioritisation process using Delphi method. *Lancet Healthy Longev.* 2022;3(1):e31–41. 10.1016/S2666-7568(21)00302-0 35028632 PMC8732284

[42] Thomson DR Rhoda DA Tatem AJ : Gridded population survey sampling: a systematic scoping review of the field and strategic research agenda. *Int J Health Geogr.* 2020;19(1): 34. 10.1186/s12942-020-00230-4 32907588 PMC7488014

[43] Khan SM Liu X Nath S : A global review of publicly available datasets for ophthalmological imaging: barriers to access, usability, and generalisability. *Lancet Digit Health.* 2021;3(1):e51–66. 10.1016/S2589-7500(20)30240-5 33735069 PMC7618278

[44] Burton MJ Ramke J Marques AP : The *Lancet Global Health* Commission on Global Eye Health: vision beyond 2020. *Lancet Glob Health.* 2021;9(4):e489–551. 10.1016/S2214-109X(20)30488-5 33607016 PMC7966694

[45] McCormick I : The Rapid Assessment of Avoidable Blindness survey: Review of the methodology and protocol for the seventh version (RAAB7). *Open Science Framework.* 2024. 10.17605/OSF.IO/UWD2E PMC11143406 38828387

