# Supplementary material for: The Rapid Assessment of Avoidable Blindness survey: Review of the methodology and protocol for the seventh version (RAAB7)
Source: Wellcome Open Res. 2024 Mar 8;9:133. [Version 1] doi: 10.12688/wellcomeopenres.20907.1 (PMC11143406; doi:10.12688/wellcomeopenres.20907.1)
Supplement: Peer review history

# Reviewer report: Reviewer response for version 1

**DOI:** 10.21956/wellcomeopenres.23133.r81729

**Published:** 2024-05-31

**Authors:** Kwadwo Owusu Akuffo

**Affiliations:** 1 Kwame Nkrumah University of Science and Technology, Kumasi, Ashanti Region, Ghana

1. OVERALL ASSESSMENT OF THE STUDY

 This method article has been carefully written. The rationale behind the development of the new RAAB protocol is well-detailed. The study's objectives are clearly explained, and the choice of the target group or population is described. The sampling and examination protocols have also been well-described and are technically sound. Potential protocol limitations were highlighted, and measures taken to address their impact on the results and findings from using the protocol were discussed. Overall, a well-written article. 

2. MANDATORY REVIEW QUESTIONS

 The rationale for developing a new Rapid Assessment of Avoidable Blindness (RAAB) methodology has been clearly explained and detailed.

 The current methodology – RAAB7 – is an improvement on the previous RAAB6 methodology. All previous methods – from RAAB4.02 to RAAB6 – used paper-based questionnaires to collect participant data. Therefore, the aim of the development of RAAB7 was to fully digitize RAAB and ensure that data collected using the protocol is available on a single, easily accessible platform, thereby optimizing eye health planning.

 Further, the RAAB7 saw the introduction of the Peek Vision software and data intelligence platform to improve the speed and accuracy of the data collection procedure. This software was an improvement on the pilot mobile application employed in mRAAB6.

 The method was well-described and technically sound.

 Ethical considerations are spelled out for the main RAAB7 project and individual RAAB surveys that will employ this methodology. The sampling protocol includes well-laid down objectives, a specific target population and the reasons for such a target group, a well-detailed sampling technique /methodology, clearly explained sample size calculations, well-defined eligibility and exclusion criteria, and a template study protocol, which includes participant information and a written consent sheet.

 The examination protocol is also well-detailed and covers vital ophthalmic and optometric tests and procedures that are ideal for meeting the objectives of the RAAB7 protocol. These tests and procedures are also very basic and can be undertaken in numerous settings, ensuring the broad applicability of the survey protocol. They include distance visual acuity measurement and classification based on the latest International Classification of Diseases (ICD)-11, external eye examination with a pen torch, fundus examination with direct ophthalmoscopy, cataract follow-up questions, and optional data collection sections on participant sociodemographics. The protocol provides extended data on the RAAB7 questionnaire, ophthalmic examination variables, response options, and definitions. The optional Diabetic Retinopathy and Disability modules further augment the RAAB7 protocol.

 The RAAB7 protocol is well-detailed and provides sufficient information to replicate its methodology easily.

 All protocols – sampling and examination protocols – are well explained, with the techniques detailed and the tools or equipment involved stated. Where there are modifications to standard optometric procedures, reasons are well-spelt out and justified.

 Also, the protocol involves a training section for individual RAAB users and their data collection team, and this training is scheduled to take place before data collection is used. This further enhances the replicability of the protocol.

 This protocol does not include a results section, as it only describes a survey method.

 The conclusions purported concerning the RAAB7 method and its performance are sufficiently backed by the findings presented in the article.

Is the rationale for developing the new method (or application) clearly explained?

Yes

Is the description of the method technically sound?

Yes

Are the conclusions about the method and its performance adequately supported by the findings presented in the article?

Yes

If any results are presented, are all the source data underlying the results available to ensure full reproducibility?

No source data required

Are sufficient details provided to allow replication of the method development and its use by others?

Yes

Reviewer Expertise:

Ophthalmic epidemiology, ocular nutrition, public health

I confirm that I have read this submission and believe that I have an appropriate level of expertise to confirm that it is of an acceptable scientific standard.

# Reviewer report: Reviewer response for version 1

**DOI:** 10.21956/wellcomeopenres.23133.r79818

**Published:** 2024-05-21

**Authors:** Xiu Juan Zhang

**Affiliations:** 1 Department of Ophthalmology and Visual Sciences, The Chinese University of Hong Kong, Hong Kong, China; 2 Department of Ophthalmology, The Hong Kong University, Hong Kong, Hong Kong, Hong Kong

I appreciate the chance to offer a peer assessment of this well-written article, " The Rapid Assessment of Avoidable Blindness survey: Review of the methodology and protocol for the seventh version (RAAB7)". RAAB is a population-based cross-sectional survey methodology used to collect data on the prevalence of vision impairment and its causes and eye care service indicators among the population 50 years and older. The current study gave an updated overview of RAAB, described the survey methodology in 2023 and highlighted priority areas for future research and development.

 The manuscript is well-organized and comprehensive. One comment I have is to include the limitations or perspectives of the RAAB protocol in the relevant section. It is worth noting that the RAAB protocol may tend to over-estimate the contribution of anterior segment conditions, such as refractive error and cataract, while potentially under-estimating the contributions of posterior segment conditions to vision impairment. This limitation arises due to the constraints of field work conditions, which may not allow for a detailed assessment of posterior segment conditions. However, the inclusion of the pupillary light reflex, particularly the evaluation of relative afferent pupillary defect (RAPD), can enhance the diagnostic accuracy of posterior segment diseases, especially in patients with severe cataracts or opaque corneas.

Is the rationale for developing the new method (or application) clearly explained?

Yes

Is the description of the method technically sound?

Yes

Are the conclusions about the method and its performance adequately supported by the findings presented in the article?

Yes

If any results are presented, are all the source data underlying the results available to ensure full reproducibility?

Yes

Are sufficient details provided to allow replication of the method development and its use by others?

Yes

Reviewer Expertise:

Ophthalmic epidemiology and refractive error

I confirm that I have read this submission and believe that I have an appropriate level of expertise to confirm that it is of an acceptable scientific standard.
